# Nematode RALF-Like 1 Targets Soybean Malectin-Like Receptor Kinase to Facilitate Parasitism

**DOI:** 10.3389/fpls.2021.775508

**Published:** 2021-12-17

**Authors:** Xin Zhang, Dongmei Wang, Jia Chen, Dousheng Wu, Xianzhong Feng, Feng Yu

**Affiliations:** ^1^State Key Laboratory of Chemo/Biosensing and Chemometrics, College of Biology, Hunan Key Laboratory of Plant Functional Genomics and Developmental Regulation, Hunan University, Changsha, China; ^2^Key Laboratory of Soybean Molecular Design Breeding, Northeast Institute of Geography and Agroecology, The Innovative Academy of Seed Design, Chinese Academy of Sciences, Changchun, China; ^3^State Key Laboratory of Hybrid Rice, Hunan Hybrid Rice Research Center, Changsha, China; ^4^State Key Laboratory of Crop Stress Adaptation and Improvement, School of Life Sciences, Henan University, Kaifeng, China

**Keywords:** RALF, malectin-like receptor kinase, root-knot nematodes (*Meloidogyne* spp.), soybean, single nucleotide polymorphism (SNP)

## Abstract

Soybean [*Glycine max* (L.) Merr. ] is one of the most strategical oilseed crops that provides sustainable source of protein and oil worldwide. Cultivation of soybean is severely affected by root-knot nematode (RKN). However, the mechanism of RKN parasitism to soybeans is largely unknown. In this study, we identify *GmLMM1*, which encodes a homolog of FERONIA-like receptor kinase in soybean, as a susceptible gene toward nematode. Mutations of *GmLMM1* exhibit enhanced resistance against the RKN *Meloidogyne incognita*. RNA-sequencing (RNA-seq) analysis reveals a similar differential expression pattern for genes regulated by *GmLMM1* (*Gmlmm1* vs. wild-type) and *M. incognita* (*M. incognita* vs. mock), supporting the role of *GmLMM1* in *M. incognita* infection. Unlike FERONIA in *Arabidopsis*, GmLMM1 specifically binds to *Mi*RALF1 and *At*RALF23 that suppress plant immunity, but not *Mi*RALF3 and *At*RALF1. Moreover, we found that the single-nucleotide polymorphism (SNP) in *GmLMM1* leads to the natural resistance against RKNs in soybeans. Collectively, these findings uncover GmLMM1 as a susceptible target of nematode RALF-like 1 and provide new genetic resource for nematode resistant breeding.

## Introduction

Plant-parasitic nematodes (PPNs) are devastating plant pathogens that infect the majority of agriculturally important crops and cause severe yield losses worldwide (Jones et al., 2013). Soybean [*Glycine max* (L.) Merrill], an important crop that provides protein and oil to mankind, is severely affected by PPNs. The economically most important nematodes that attack soybean are sedentary endoparasitic nematodes such as cyst nematodes and root-knot nematodes (RKNs). Soybean nematode diseases are devastating because the aboveground disease symptoms are normally only visible at later stages of infection when the nematode is hard to be eradicated by chemical pesticides and a huge damage has already occurred (Shaibu et al., 2020). Therefore, there is an increasing demand to understand the virulence mechanism of nematode infection, identify new resistance resources against nematodes, and engineer new soybean cultivars with durable nematode resistance.

Plant innate immunity is a major form of plant resistance and has great potential for disease control. Plant innate immunity relies on the recognition of pathogen-associated molecular patterns (PAMPs) by cell surface localized pattern recognition receptors (PRRs) (Jones and Dangl, 2006; Zhou and Zhang, 2020). The activation of PAMP-triggered immunity (PTI) is accompanied by cell wall reinforcement, induction of defense-responsive genes, reactive oxygen species (ROS) burst, and mitogen-associated protein kinase (MAPK) accumulation (Böhm et al., 2014; Macho and Zipfel, 2014). PRRs consist of receptor-like kinases (RLKs) and receptor-like proteins (RLPs). Recent studies have indicated that PAMP perception by PRRs requires coreceptors (Tang et al., 2017; Zhou and Zhang, 2020). For example, the PRR EF-TU receptor (EFR) and flagellin sensing 2 (FLS2), which are receptors for the bacterial elongation factor Tu epitope elf18 and flagellin epitope flg22, respectively, require the somatic embryogenesis receptor-like kinase (SERK) family of RLKs such as BRI1-associated kinase 1 (BAK1) as coreceptors for PAMP recognition. In addition, some RLKs containing distinct ectodomains can also interact with PRRs and regulate PTI. The malectin-like receptor kinase FERONIA (FER) from the *Catharanthus roseus* receptor-like kinase-1-like (*Cr*RLK1L) family can facilitate the PAMP-induced FLS2-BAK1 and EFR-BAK1 complex formation to initiate immune signaling (Stegmann et al., 2017). Interestingly, ANXUR1 and ANXUR2, two other *Cr*RLK1L family members, can also interact with FLS2 and negatively regulate FLS2-mediated immune response (Mang et al., 2017). Therefore, the PRRs can interact with different coreceptors and regulate plant innate immunity.

Rapid alkalinization factors (RALFs) are ligands of *Cr*RLK1L receptors. RALFs were known for their ability to induce rapid alkalinization of the extracellular compartment of plant cells. The RALF–FER interaction regulates a series of developmental and stress responses (Zhang et al., 2020b). For example, RALF1 from *Arabidopsis* binds to the extracellular domain of FER and initiates FER kinase activity inhibiting H^+^-ATPase activity and root elongation (Haruta et al., 2014). RALF-FER also plays an important role in plant immunity regulation. Several RALFs including RALF23 and RALF33 inhibit plant immunity by destabilizing FER-facilitated immune complex formation between immune receptors FLS2 and EFR with their coreceptors BAK1 (Stegmann et al., 2017). Lorelei-like glycosylphosphatidylinositol-anchored protein 1 (LLG1) functions as a coreceptor of FER and modulates FLS2 accumulation regulating plant immunity (Shen et al., 2017). Interestingly, RALF23 can be perceived by LLG2 and induces a complex between FER and LLG2 to regulate immune signaling (Xiao et al., 2019). In addition, FER phosphorylates and destabilizes MYC2, the master regulator of jasmonic acid (JA) signaling, to positively modulate immunity, whereas RALF23 stabilizes MYC2 through FER and negatively contributing to plant immunity (Guo et al., 2018).

Though RALFs are initially found and widely distributed in plants, recent studies have identified several RALF-likes in pathogens (Masachis et al., 2016; Thynne et al., 2017; Blackburn et al., 2020; Zhang et al., 2020a). The RALF-like from *Fusarium oxysporum* enhances fungi virulence, and Arabidopsis plants lacking FER are more resistant against *F. oxysporum* (Masachis et al., 2016). This indicates that the RALF-like from the pathogen mimics the function of plant RALFs and is perceived by plant FER suppressing host immunity. Nematodes also encode and secrete RALF-likes into host cells during parasitism. These RALF-likes are highly expressed during the parasitic stages of nematode infection. Similar to plant RALFs, nematode RALF-likes directly bind to the extracellular domain of FER and inhibit immune responses facilitating nematode infection (Zhang et al., 2020a). Though the role of nematode RALF-likes has been studied in *Arabidopsis*, the practical roles of nematode RALF-likes on crops remain unknown.

In this study, we screened a soybean ethyl methane sulfonate (EMS) library for mutants that are resistant to the RKN *Meloidogyne incognita* (*M. incognita*). We identified two mutants, named resistant to *M. incognita* 1/2 (*rmi1/2*), showed significantly enhanced resistant to *M. incognita*. Positional cloning revealed that *rmi1/2* have mutations in the same gene, *Glyma.13G054400* or *GmLMM1*, which was recently shown to encode a malectin-like receptor kinase and show high sequence similarity with *Arabidopsis FER* (Wang et al., 2020). We further confirmed that nematode-encoded RALF-like 1 targets GmLMM1 inhibiting immune response of soybean and promoting nematode infection. Natural variations in *GmLMM1* confer enhanced disease resistance against *M. incognita*. Our results provide novel insight into nematode–soybean interactions and also new genetic resources for resistant breeding.

## Materials and Methods

### Plant Materials and Growth Conditions

Soybean cultivar “Williams 82” and “DN50” obtained from the Chinese Academy of Agricultural Sciences were used as WT for all soybean assays. *Gmrmi* mutants (including *Gmrmi1* and *Gmrmi2*) were mutagenized from the soybean treated with 5% EMS (Wang et al., 2020). All seedlings of soybean were grown in the field from May to October for separate group construction and gene mapping, or in a climate chamber, whose temperature was maintained at 25°C and humidity at 50% with a 14-h photoperiod for nematode infection assays.

### Genetic Mapping of *GmLMM1*

We performed the detailed assay of mapping the *GmLMM1* gene as the method described by Wang (Wang et al., 2020).

### Nematode Infection Assays

For nematode infection assays toward soybean, 1-mL aliquot of a suspension containing 500 *M. incognita* pre-J2s was inoculated on the roots of 14-day-old wild-type and mutation soybean at 25°C. Subsequently, the number of nematodes inoculated was recorded at different stages. Fuchsin-stained roots were observed with an Olympus BX53 microscope (Olympus, Japan). For each experiment, 10–20 plants were used per treatment, and each experiment was repeated independently at least three times.

### RNA-seq Analysis

RNA-sequencing was performed by Novogene (Beijing, China). Soybean cultivar “Williams 82” and *Gmlmm1-1* were grown for 14 days and then the roots of which were inoculated with 1-mL aliquot of a suspension containing 500 *M. incognita* pre-J2s. Seven days post inoculation, the roots of soybean were collected and a total amount of 1 μg RNA per sample was used as input material for the RNA-sequencing (RNA-seq) library preparations. Briefly, mRNA was purified from total RNA using poly-T oligo-attached magnetic beads. RNA degradation and contamination were monitored on 1% agarose gels. RNA purity was checked using the NanoPhotometer spectrophotometer (IMPLEN, CA, USA). RNA integrity was assessed using the RNA Nano 6000 Assay Kit of the Bioanalyzer 2100 system (Agilent Technologies, CA, USA). Sequencing libraries were generated using NEB Next UltraTM RNA Library Prep Kit for Illumina (NEB, USA) following the manufacturer's recommendations, and index codes were added to attribute sequences to each sample. The clustering of the index-coded samples was performed on a cBot Cluster Generation System using TruSeq PE Cluster Kit v3-cBot-HS (Illumia) according to the manufacturer's instructions. After cluster generation, the library preparations were sequenced on an Illumina NovaSeq platform, and 150-bp paired-end reads were generated. Raw data (raw reads) of fastq format were first processed through in-house perl scripts. In this step, clean data (clean reads) were obtained by removing reads containing adapter, reads containing ploy-N, and low-quality reads from raw data. Reference genome and gene model annotation files were downloaded from genome website directly. Index of the reference genome was built using Hisat2 v2.0.5, and paired-end clean reads were aligned to the reference genome using Hisat2 v2.0.5. The fragments per kilobase of exon model per million reads mapped (FPKM) value of each gene was calculated using cuff links (Trapnell et al., 2012), and the read counts of each gene were obtained using htseq-count. DEGs were identified using the DESeq estimating function and nbinom test. Notably, *p-*value <0.05 and fold changes >2 or <0.5 were used as the criteria for identifying significant DEGs. A hierarchical clustering analysis of the DEGs was performed to explore gene expression patterns. The GO enrichment of the DEGs was estimated using the DESeq R package based on the hypergeometric distribution.

### Expression and Purification of RALF/RALF-Like Peptides

The DNA sequences of the bioactive form of the *At*RALF1 peptide (amino acids 72–120) and *At*RALF23 peptide (amino acids 89–138) were PCR-amplified from *Arabidopsis* cDNA, and *MiRALF1/3* were amplified from *M. incognita* cDNAs *via* PCR (Zhang et al., 2020a). All the *RALF/RALF-like* genes were constructed into a modified expression vector, pET-28a, with a 6 × His-MBP tag. Then, the constructed vector was transformed into the *E. coli* strain BL21 Gold (DE3) and cultured in LB medium overnight at 37°C, before transferred to fresh media at a 1:100 dilution. When the OD_600_ value of bacteria solution nearly reached 0.5, a final concentration of 0.5 mM isopropylthio-β-D-1-galactopyranoside (IPTG, Sangon Biotech, Shanghai, China) was used and the cultures were incubated at 28°C for 4 h to induce expression. Subsequently, RALF/RALF-like proteins with the 6 × His-MBP tag were expressed and collected by centrifugation for purification. One volume of the collected cells was resuspended in seven volumes of lysis buffer [50 mM Tris-HCl (pH 8.0), 300 mM NaCl, and 10 mM imidazole] supplemented with 1 mM of the protease inhibitor PMSF (Selleck Chemicals, Houston, USA) and suspended for sonication. The extract was centrifuged at 10,000 × g for 15 min at 4°C, the soluble fraction was obtained, and the desired protein was bound to His-beads (Ni Sepharose HP, GE Healthcare) overnight. Subsequently, the beads were washed sequentially with seven volumes of wash buffer 1 (50 mM Tris-HCl, 300 mM NaCl, and 20 mM imidazole, pH 8.0), wash buffer 2 (50 mM Tris-HCl, 300 mM NaCl, and 50 mM imidazole, pH 8.0), and wash buffer 3 (50 mM Tris-HCl, 300 mM NaCl, and 100 mM imidazole, pH 8.0) for 10 min each prior to eluting the 6 × His-MBP-RALF protein with elution buffer (50 mM Tris-HCl, 300 mM NaCl, and 250 mM imidazole, pH 8.0) for 3 h at 4°C.

### GST Pull-Down Assay

The GmLMM1 extracellular domain (from residues 27 to 452, GmLMM1^ECD^) was amplified and inserted into the vector pGEX-4T-1 using the primers GmLMM1-ECD-F and GmLMM1-ECD-R (Supplementary Table S1) to produce a GST-GmLMM1^ECD^ fusion protein in the *E. coli* strain BL21 Gold (DE3). Cells containing GST-GmLMM1^ECD^ were collected by centrifugation. One volume of pelleted cells was resuspended in seven volumes of GST lysis buffer [50 mM Tris-HCI (pH 8.0), 100 mM NaCl, 10 mM MgCl_2_, 1 mM dithiothreitol (DTT), and 10% glycerol] supplemented with a 1 × solution of the protease inhibitor PMSF and suspended for sonication. After centrifugation (4000 rpm, 4°C, 15 min), the supernatant was absorbed onto prewashed GST agarose (Glutathione Sepharose 4B, GE Healthcare) overnight. Subsequently, the agarose was washed with GST wash buffer (50 mM Tris-HCI, 150 mM NaCl, 10 mM MgCl_2_, 1 mM DTT, 10% glycerol and 0.1% Triton X-100, pH 8.0) three times for 10 min prior to eluting the fusion protein for 2 h in GST elution buffer (25 mM Tris-HCl, 20 mM glutathione, 100 mM NaCl, 10 mM MgCl_2_, 1 mM DTT, and 10% glycerol, pH 7.5). For GST pull-down assays, the agarose beads were prewashed three times with 1 mL of GST-binding buffer (20 mM HEPES, 40 mM KCl, and 1 mM EDTA, pH 7.5). The GST-GmLMM1^ECD^ fusion protein and GST protein were separately mixed with *At*RALF1, *At*RALF23, and *Mi*RALF1/3 and then incubated with the prewashed agarose beads (20 μL) in 400 μL of GST-binding buffer at 4°C overnight. Subsequently, the mixture was separated by centrifugation at 500 × g for 1 min, and the precipitate was washed three times with GST wash buffer for 10 min. The washed precipitate was then eluted with 50 μL of GST elution buffer for 2 h. After low-speed centrifugation, the eluate was mixed with SDS loading buffer, boiled, and analyzed using SDS-PAGE and immunoblotting with a GST-Tag (12G8) Mouse mAb (Abmart, Shanghai, China) and a His-Tag (2A8) Mouse mAb (Abmart, Shanghai, China). GST pull-down assays for other RALF proteins were performed using the same procedure.

### Coimmunoprecipitation Assay

Proteins were extracted from 7-day *GmLMM1-HA* transgenic plants (Wang et al., 2020) using NEBT buffer, incubated with *At*RALF23 or *Mi*RALF1 at a concentration of 1 μM. After immunoprecipitation with HA beads (Bimake, Houston, USA) overnight at 4°C, the beads were washed three times with NEBT buffer supplemented with 1% TritonX-100 for 15 min at 4°C and then boiled with protein loading buffer. GmLMM1-HA was tested using an anti-HA antibody [HA-Tag (C29F4) Rabbit mAb], and RALFs-His was tested by His-Tag (2A8) Mouse mAb.

### Docking Experiments

GmLMM1^ECD^ and GmLLG2 were docked onto the 3D structure of the RALF23-LLG2-FER^ECD^ complex (PDB code: 6A5E) by mutating the corresponding residues of FER^ECD^ and LLG2 in COOT (Emsley and Cowtan, 2004), guided by sequence alignment of GmLMM1^ECD^ with FER^ECD^ and GmLLG2 with LLG2. In the final models, no residues fell in the disallowed regions in the Ramachandran plot, and the complex was perfectly assembled with no obvious clashes in individual interfaces. Structural images were prepared in PyMOL (The PyMOL Molecular Graphics System, version 1.7 Schrödinger, LLC). The structure prediction was performed *via* AlphaFold (https://www.alphafold.ebi.ac.uk).

### Phylogenetic Analysis

Amino acid sequences of GmLMM1 and GmRALFs homologs were obtained by a BLASTP search against Phytozome (version 12.1; http://www.phytozome.net). The phylogenetic analysis was performed as described previously with some modifications (Wang et al., 2020).

### Statistical Analysis

Statistical significance was determined by conducting Student's *t*-tests or one-way ANOVA using SPSS 23.0 software (SPSS).

## Results

### Mutation of *GmLMM1* Enhances Soybean Resistance Against *M. incognita*

**Figure 1:**
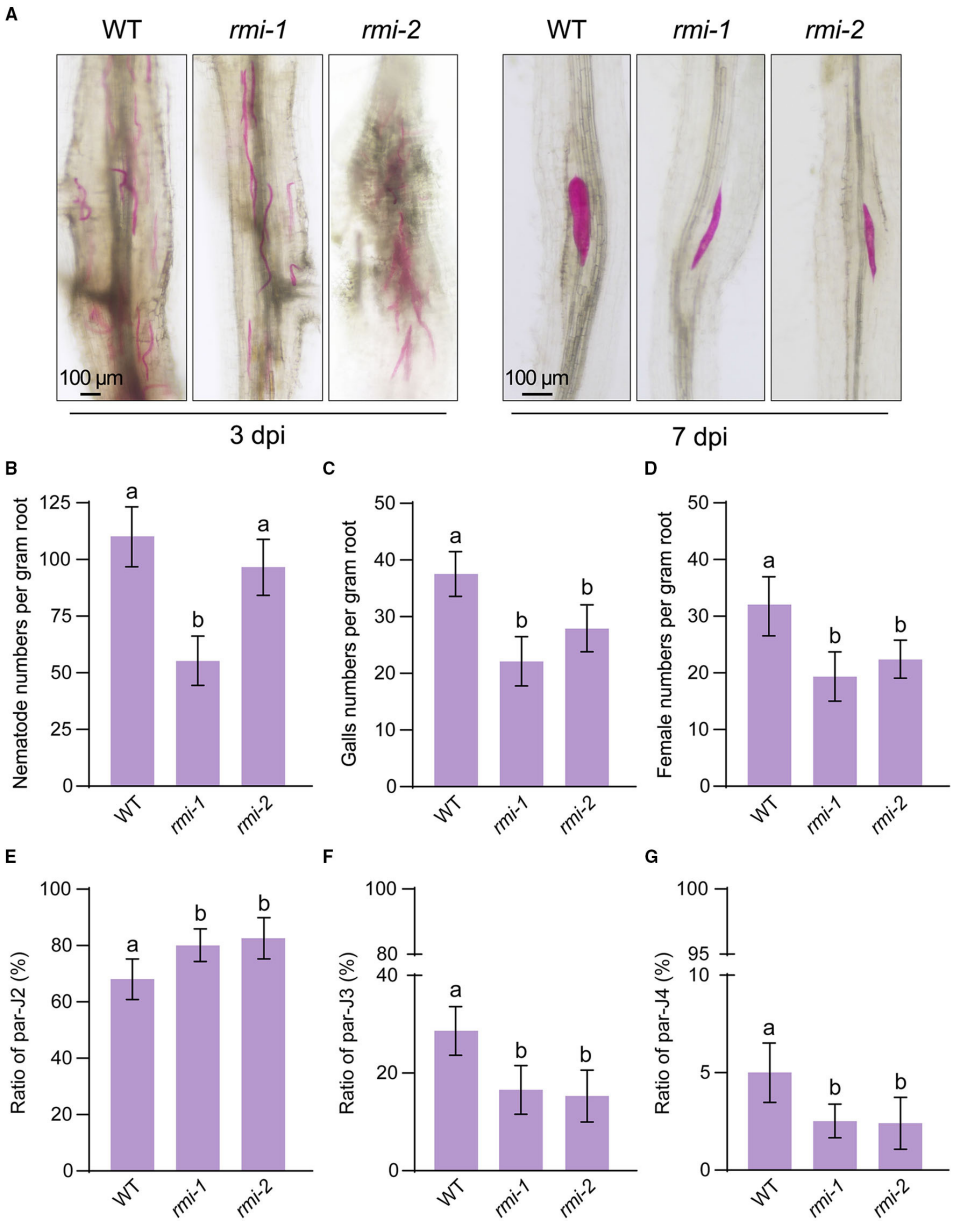
Two soybean EMS mutants showed enhanced resistance to *M. incognita*. **(A)** Representative images of fuchsin-stained *M. incognita* parasites on wild-type and *rmi1/2* soybean roots at 3 and 7 days postinfection (dpi). **(B)** *M. incognita* parasites numbers per gram of soybean roots at 3 dpi. **(C, D)** *M. incognita* gall (**C**) and female (**D**) numbers per gram of soybean roots at 30 dpi. **(E–G)** Statistical analysis of *M. incognita* parasites developmental stages consisting of par-J2 **(E)**/ par-J3 **(F)**/ par-J4 **(G)** nematodes to the total parasitic nematodes at 14 dpi. At least three biological replicates of **(B–G)** were performed, and the similar results were obtained. Data are presented as the mean ± S.D., *n* ≥ 10; one-way ANOVA (different letters represent *p* < 0.05).

To identify genes that negatively regulate soybean resistance against *M. incognita*, we inoculated 600 M2 seedlings that were mutagenized with EMS (Wang et al., 2020) and screened for *M. incognita* resistant mutants. We found that two mutants were more resistant against *M. incognita* compared with wild-type soybean (Figures 1A,B), which were named *resistant to M. incognita 1* (*rmi1*) and *rmi2*, respectively. Precisely, there were significant differences in the nematode number and nematode development between *rmi1/2* and wild-type plants. At 3 days post infection (dpi), the number of nematodes was significantly lower in the *rmi1* mutant compared with that in the wild-type plants, but no differences in *rmi2* (Figures 1A,B). At 7 dpi, most of the nematodes parasitizing wild-type plants turned to the parasitic third stage (par-J3, morphologically thick and short), whereas the nematodes in both *rmi1/2* still remained in the parasitic second stage (par-J2 stage, morphologically thin and long) (Figure 1A). Subsequently, at 30 dpi, most of the nematodes reached the reproduction stage, the features of which is the formation of galls. Notably, the galls number in *rmi1/2* was significantly lower compared with wild-type plants (Figure 1C). We also calculated female RKNs (morphologically round basal knobs) in *rmi1/2* and wild-type plants and found a significant decrease in *rmi1/2* compared with wild-type plants (Figure 1D). Since the obvious differences in developmental stages ranging from par-J2 to the parasitic fourth stage (par-J4), statistical analysis of the developmental stage ratio was conducted at 14 dpi. The ratio of nematodes in par-J2 was <70% in wild-type plants compared to nearly 80% in *rmi1/2* (Figure 1E). Moreover, the ratio of nematodes in par-J3 almost reached 30% in wild-type plants, while it only reached 15% in *rmi1/2* (Figure 1F). Similarly, the ratio of nematodes in par-J4 was nearly 5% in wild-type plants and only 2% in *rmi1/2* (Figure 1G). Collectively, there was a significant developmental delay ranging from par-J2 to par-J4 of *M. incognita* in *rmi1/2* mutants compared with wild-type plants, suggesting that *rmi1/2* mutants were more resistant against *M. incognita* parasitism.

Interestingly, *rmi1* and *rmi2* mutants have not only nematode resistance phenotype, but also spontaneous lesions on their leaves, namely *Gmlmm1-1* and *Gmlmm1-2* in the recent article Wang et al. (2020) we published. Thus, *rmi1* and *rmi2* carried two different mutation types in the same predicted gene, *Glyma.13G054400* (*GmLMM1*). *rmi1* (*Gmlmm1-1*) had a C to T substitution within the coding region of *Glyma.13G054400*, leading to an early stop codon and thus an incomplete protein (Supplementary Figure S1A). Whereas *rmi2 (Gmlmm1-2)* harbors a T to A substitution in *Glyma.13G054400*, this substitution only changed the amino acid from histidine to leucine at position 407 (Supplementary Figure S1A) (Wang et al., 2020). For consistency, we named the gene-controlling *rmi1* and *rmi2* nematode-resistant phenotypes *GmLMM1* as previously described (Wang et al., 2020).

**Figure 2:**
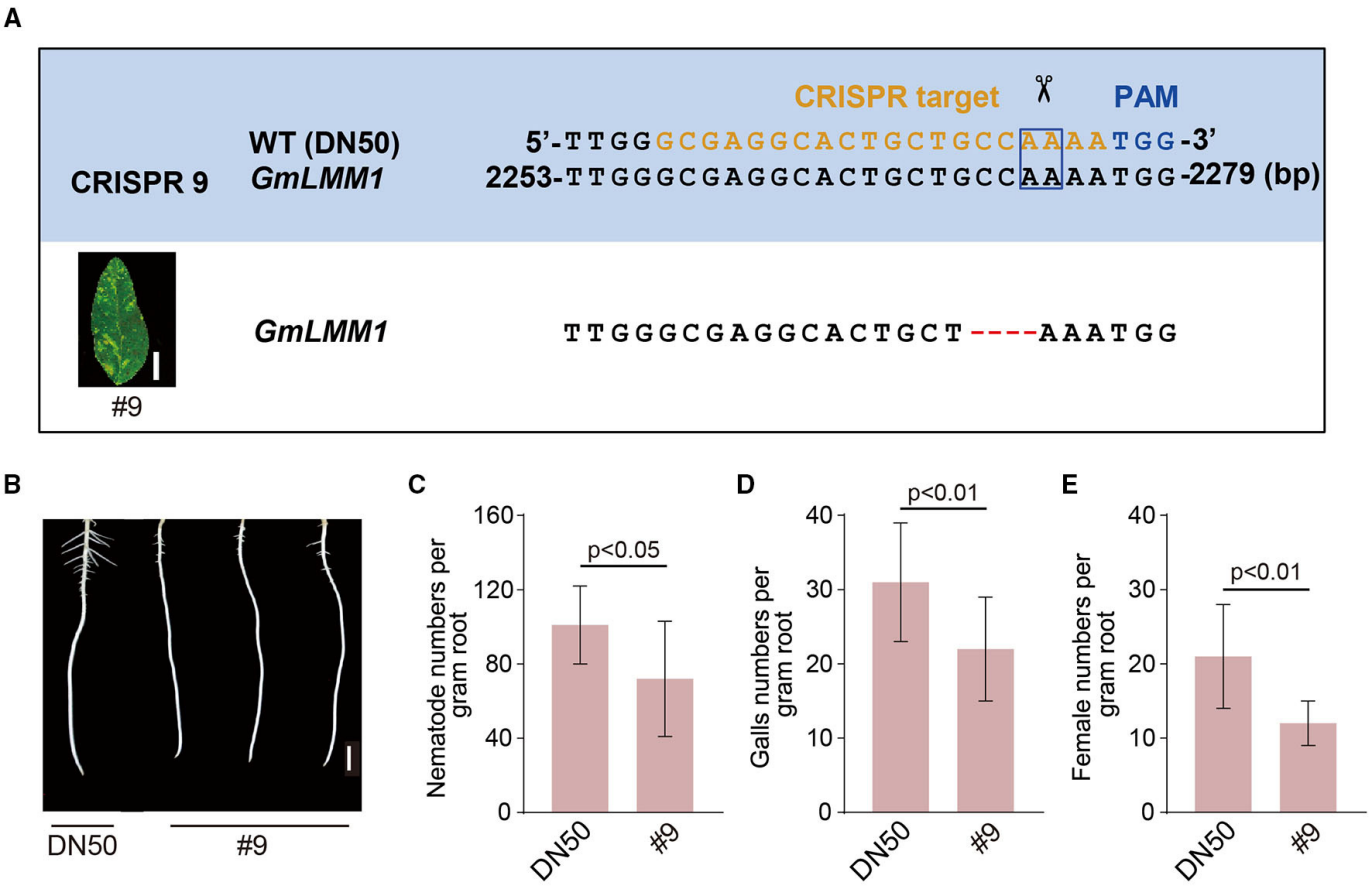
Editing of the GmLMM1 causes the resistance to *M. incognita*. **(A)** Editing of GmLMM1 by CRISPR/Cas9. The CRISPR target sequence is shown in orange, and the corresponding protospacer adjacent motif (PAM) site is shown in blue. The box indicates the putative sites shear cut by cas9. **(B)** Phenotype appearance of the line #9 mutant root. Scale bar presents 1 cm. **(C)** *M. incognita* parasites numbers per gram of soybean roots at 7 dpi. **(D,E)** *M. incognita* gall **(D)** and female **(E)** numbers per gram of soybean roots at 30 dpi. At least three biological replicates of **(C–E)** were performed, and the similar results were obtained. Data are presented as the mean ± S.D., *n* ≥ 10; Student's *t*-test.

*GmLMM1* encodes a RLK that contains a malectin-like domain in its extracellular domain, a transmembrane domain, and an intracellular kinase domain in its C-terminal region (Supplementary Figure S1A). The closest homology of GmLMM1 in *Arabidopsis* is the *Cr*RLK1L FER which shares a high degree of amino acid identity, particularly in the extracellular domain (Supplementary Figures S1B, S2). To confirm the role of *GmLMM1* in regulating nematode resistance, we generated heritable mutation in the coding sequence of *GmLMM1* by CRISPR-Cas9 mutagenesis (Wang et al., 2020). Among them, line #9 harbored a 4-bp deletion in *GmLMM1* (Figure 2A) and showed an autoimmunity-related root phenotype (Figure 2B). Thus, line #9 was selected to further confirm the role of *GmLMM1* in the nematode parasitism. Indeed, there were significant differences in the nematode infection number and nematode development between line #9 and wild-type plants (DN50). At 7 dpi, the number of nematodes was significantly lower in line #9 compared with that in the wild-type plants (Figure 2C). Moreover, at 30 dpi, the formation of galls in line #9 was repressed (Figure 2D), and the female number in line #9 was significantly reduced compared with wild-type plants (Figure 2E). Altogether, these results demonstrate that *GmLMM1* negatively regulates nematode resistance.

### *GmLMM1*-Regulated Genes Are Involved in Nematode Susceptibility

**Figure 3:**
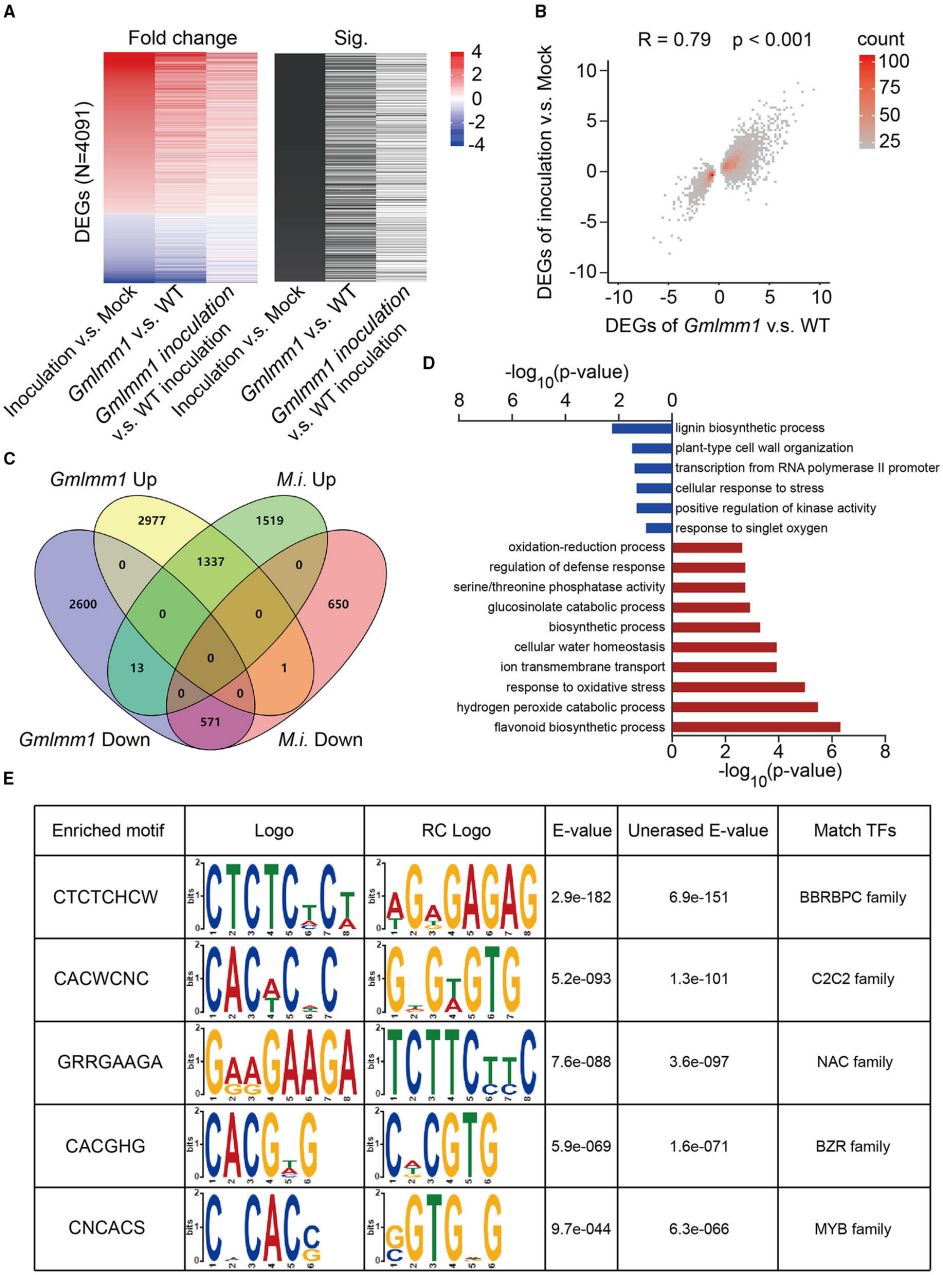
RNA-seq assay identifies *GmLMM1*-regulated genes in defense response against *M. incognita*. **(A)** Heatmap of fold changes in DEGs following *M. incognita* inoculation (*n* = 3) or *GmLMM1* mutation or the combination of both. A significant difference in *p* < 0.05 was observed. **(B)** Spearman's correlation analysis of DEGs in plants following *M. incognita* inoculation (*n* = 3) compared with *GmLMM1* mutation (*n* = 3). **(C)** Venn diagram showing overlaps between genes upregulated (*Gmlmm1* Up) or downregulated (*Gmlmm1* Down) in *Gmlmm1* mutant compared with WT and *M. incognita*-induced (*M.i*. Up) or *M. incognita*-repressed genes (*M.i*. Down) under the infection of *M. incognita* or not in WT. **(D)** GO enrichment analysis based on the *p*-values for similarly expressed genes between groups subjected to the overlap genes of up genes (stained in red) or down genes (stained in blue). **(E)** Motif- GO enrichment analysis based on the promoters of the overlaps between *GmLMM1* and *M. incognita-* regulated genes.

To understand how *GmLMM1* negatively regulates soybean resistance at the transcriptional level, we performed RNA-seq on roots of wild-type plants and *Gmlmm1-1* with or without *M. incognita* infection. We observed profound changes in the plant gene expression profile after infection of *M. incognita* in wild-type plants or after *GmLMM1* mutation. A total of 4, 091 differentially expressed genes (DEGs) (*p* < 0.05, fold change >2 or fold change < 0.5) were detected after infection with *M. incognita* in wild-type plants (Figure 3A). Intriguingly, a proportion of the DEGs that detected in wild-type plants under the infection of *M. incognita* showed similar differential expression pattern after *GmLMM1* mutation (*p* < 0.05, Figure 3A). Clustering analysis showed that the expression changes of most *GmLMM1* and nematode infection-regulated genes were correlated (*r* = 0.79, *p* < 0.001, Figure 3B). Notably, most DEGs observed seem to be unchanged in *Gmlmm1-1* after *M. incognita* infection (Figure 3A), indicating the key role of *GmLMM1* in regulation of gene expression in response to nematode infection. Furthermore, Venn diagrams were conducted to explore the intersection of *GmLMM1* mutation and nematode infection-regulated genes. Compared with in wild-type soybean, 4,315 DEGs were upregulated and 3,184 DEGs were downregulated in *Gmlmm1* mutants, which are marked with yellow or purple, respectively (Figure 3C). Meanwhile, 2869 DEGs were upregulated and 1,222 DEGs were downregulated in wild-type soybean after *M. incognita* inoculation, which were marked with green or pink, respectively (Figure 3C). Nearly half of DEGs detected in wild-type plants after *M. incognita* inoculation were observed in the DEGs detected after *GmLMM1* mutation, among of which 1,337 DEGs were intersected upon the groups of *Gmlmm1* Up and *M.i*. Up and 571 DEGs were intersected upon the groups of *Gmlmm1* Down and *M.i*. Down (Figure 3C). Gene ontology (GO) analysis revealed that the top enriched GO processes of both upregulated and downregulated intersection DEGs, such as defense response-associated processes, cell wall organization, and response to biotic stimulus (Figure 3D). Notably, previous studies have shown that these processes play an important role in defensing nematode infection (Wang et al., 2019; Zhang et al., 2020a), suggesting that *GmLMM1* is indeed involved in transcriptional regulation of soybean resistance against *M. incognita*. To further get insight into *GmLMM1*-regulated genes, we selected the intersected DEGs above and performed DNA-binding motifs prediction in their promoters (Figure 3E). Based on the predicted binding motifs, we searched for transcription factors that recognize such DNA-binding motifs and further predicted their potential downstream targets of these transcription factors. GO enrichment analysis showed that the predicted target genes are mostly involved in JA response, salicylic acid response, and ethylene-activated signaling pathway (Figure 3E; Supplementary Figure S3), all of which are key immune signaling pathways against nematodes (Nahar et al., 2011; Martinez-Medina et al., 2017; Wang et al., 2019). Taken together, RNA-seq analysis revealed that mutation of *GmLMM1* transcriptionally upregulates defense-associated genes to enhance nematode resistance.

### RALF-Like 1 From *M. incognita* Interacts With GmLMM1

**Figure 4:**
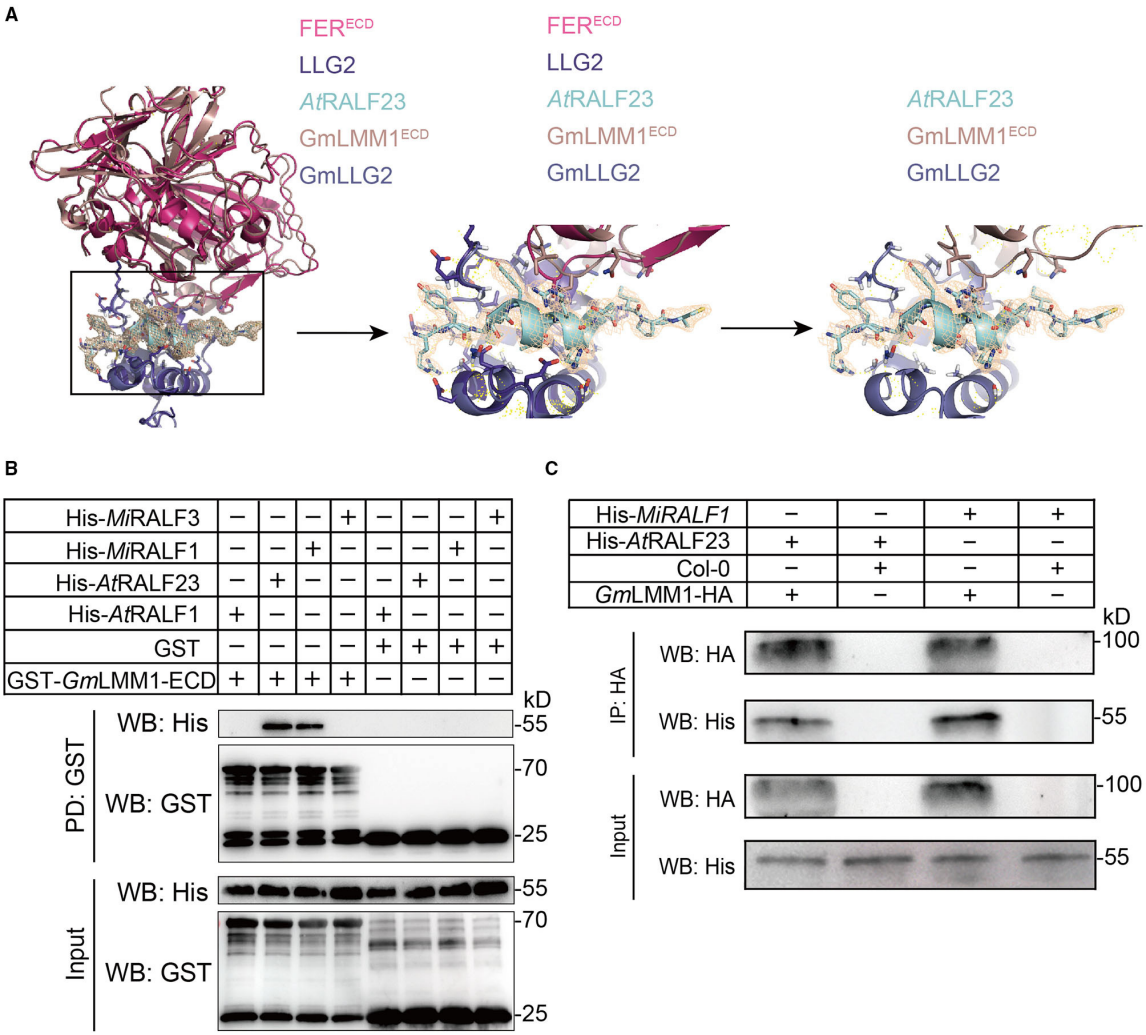
The binding assay of plant or nematode RALFs to GmLMM1. **(A)** The 3D structure of the RALF23-LLG2 (GmLLG2)/FER^ECD^ (GmLMM1^ECD^) complex. RALF23, LLG2, and FER^ECD^ are colored light cyan, dark blue, and rose red, respectively. The 3D structure of GmLLG2 and *Gm*LMM1^ECD^ was conducted by homologously modeling in COOT. The yellow mesh represents the 2Fo-Fc electron density map of RALF23 contoured at 1.0 σ. **(B)** GST pull-down assays. Different RALFs bind to GST-LMM1^ECD^. The upper panel was pulled down by GST beads, and the lower panel was input. **(C)** Co-IP assays of GmLMM1 with RALF/RALF-likes and *GmLMM1-HA* transgenic plants were used, and RALF/RALF-likes peptides were produced from *E. coli* strain BL21 Gold (DE3). All experiments were replicated at least three times with similar results.

Having ascertained that *GmLMM1* regulates nematode resistance, we sought to understand the molecular mechanism behind it. Our recent work identified several RALF-likes from *M. incognita* (*Mi*RALFs) and found that *Mi*RALFs can mimic the function of plant RALF by interacting with *Arabidopsis* FER, inhibiting plant immunity and facilitating nematode parasitism (Zhang et al., 2020a). Given that GmLMM1 is a homology of FER, we wondered whether *Mi*RALFs can also interact with GmLMM1. It has been shown that the N-terminal region of *Arabidopsis* RALF23 is perceived by LLG2 to nucleate the assembly of RALF23-LLG2-FER complex (Xiao et al., 2019). To test whether GmLMM1 shares a similar RALF23-LLG2-FER-binding mode, the extracellular domain (ECD) of GmLMM1 and GmLLG2 was docked onto the 3D structure of the RALF23-GmLLG2-GmLMM1 complex. The result showed that GmLMM1^ECD^ finely matched the electron density of FER^ECD^ without causing significant steric clashes in their models (Figure 4A). Next, we expressed four RALFs (*At*RALF1/23 and *Mi*RALF1/3) and GmLMM1^ECD^ with N-terminal His-Tag or GST-Tag, respectively, in *E. coli* and purified the proteins. Pull-down assay indicated that only *At*RALF23 and *Mi*RALF1 were pulled down with the ECD of GmLMM1 (Figure 4B). Notably, *At*RALF1 did not interact with GmLMM1. To validate the interaction between RALFs and GmLMM1 in planta, we generated HA-labeled GmLMM1 transgenic *Arabidopsis thaliana* lines and coimmunoprecipitation (Co-IP) assay was conducted. The result showed that *At*RALF23 and *Mi*RALF1 indeed can interact with GmLMM1 in planta (Figure 4C). Meanwhile, there were about 24 RALFs in soybean, and 2 RALFs clustered together with AtRALF23 (Supplementary Figure S4). GST pull-down assay showed that Glyma.07G247500 and Glyma.17G026400 clustered together with AtRALF23 could bind to GmLMM1^ECD^ (Supplementary Figure S5). Furthermore, we have conducted the infection assays after exogenous MiRALF1 and AtRALF23 peptides treatment in Williams 82 and *Gmlmm1-1*. The result showed that exogenous MiRALF1 treatment enhanced RKN parasitism 3 dpi, whereas this promotion was abolished in Gmlmm1-1 mutant, suggesting that this process depends on GmLMM1 (Supplementary Figure S6). However, exogenous AtRALF23 treatment had no significant impact on nematode infection (Supplementary Figure S6). Taken together, these results suggest that *At*RALF23 and *Mi*RALF1 potentially work as the ligands of the RLK GmLMM1, which also echoes the sight of our recent work (Zhang et al., 2020a). We assume that targeting of GmLMM1 by *Mi*RALF1 suppresses soybean immunity and promote nematode infection.

### Natural Variation of *GmLMM1* Confers Resistance Against *M. incognita*

**Figure 5:**
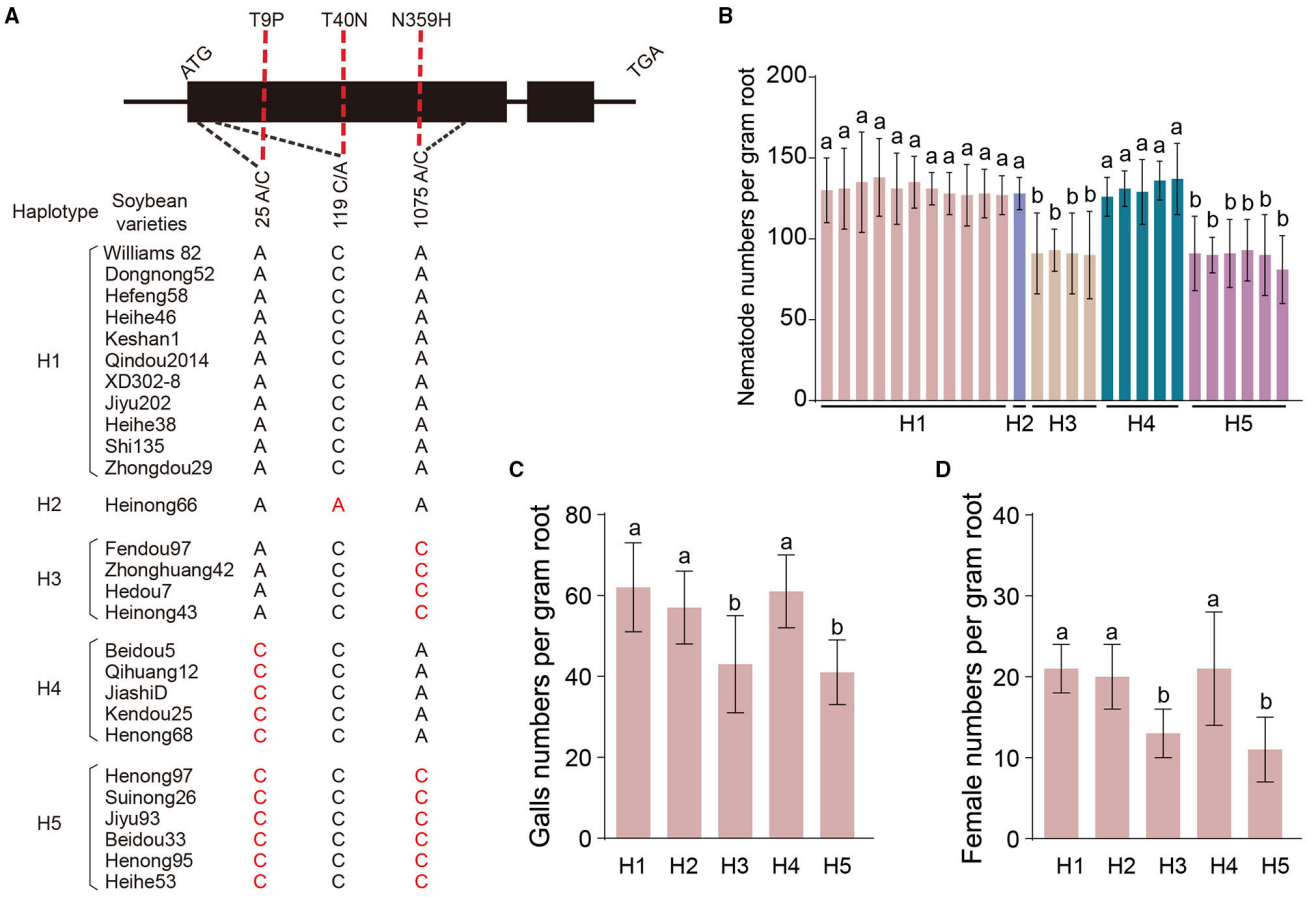
Natural variation of *GmLMM1* confers resistance against *M. incognita*. **(A)** Haplotypes identified at GmLMM1 in 27 soybean varieties. **(B)** *M. incognita* parasites numbers per gram of soybean roots at 7 dpi. **(C,D)** *M. incognita* gall **(C)** and female **(D)** numbers per gram of soybean roots at 30 dpi. At least three biological replicates of **(B–D)** were performed, and the similar results were obtained. Data are presented as the mean ± S.D., *n* ≥ 10; one-way ANOVA (different letters represent *p* < 0.05).

To investigate the relationship between *GmLMM1* and soybean resistance against *M. incognita*, we examined the variation in the coding region of *GmLMM1*. Four SNPs were identified in the coding sequence of *GmLMM1*, and three of them directing amino acid substitutions (p. T9P, p. T40N, and p. N359H) (Figure 5A, Supplementary Table S2). Based on the three nonsynonymous mutation, five haplotypes (H1-H5) were defined among 232 soybean varieties (Supplementary Table S2). To further investigate which *GmLMM1* haplotype was related to the resistance against *M. incognita*, 27 cultivars were selected as representatives of the indicated five haplotypes for further nematode infection assays (Figures 5A–D). A total of 11 varieties, including Williams 82, belong to H1, which shows no difference in the three SNPs of *GmLMM1* compared with reference sequence (*Glycine max W82.a2.v1*) (Figure 5A, Supplementary Table S2). After inoculation with *M. incognita*, the varieties belong to H3 or H5 showed enhanced resistance against *M. incognita*. In additional, the numbers of nematode parasitizing on H3 or H5 haplotypes were significantly lower compared with the other three haplotypes (Figure 5B). Furthermore, the representative varieties of H1 to H5 were selected to calculate the galls and female number. At 30 dpi, the galls in H3 and H5 plants were significantly lower than other varieties (Figure 5C), and the numbers of female RKNs in H3 and H5 lines were also significantly reduced at 30 dpi (Figure 5D), suggesting that the cultivars carried haplotypes 3 or 5 would improve the resistance against RKN. In addition, we test the polymorphisms of the three SNPs in *GmLMM1* contribution to the resistance to RKN. Of these, the p. N359H substitution in particular affects obviously to the resistance to RKN (Supplementary Figure S7), implying that the SNP played an important role for soybean resistance against to RKNs. Overall, these results suggest that *GmLMM1* is essential for the parasitism of *M. incognita* in soybean.

## Discussion

Soybean is an important crop that provides sustainable source of protein and oil worldwide. PPNs hindered the sustainable development of soybean crops, due to the extensive and continuous parasitism. Presently, more sights are focused on the cyst nematode, which is one kind of PPNs feeding on the root of soybean and is a major constraint to soybean production. The molecular mechanism of cyst nematode parasitism has become gradually clear. RKNs, another kind of PPNs sharing the similar habit with cyst nematode, also cause devastating damage on soybeans per year (Abad et al., 2008); however, the molecular mechanism underlying parasitism is largely unknown. In this study, we report two EMS-mutagenized soybean plants with increased resistance to RKN *M. incognita*. Using the genetic mapping, *GmLMM1* (*Glyma.13G054400*) was identified as the susceptible gene against *M. incognita*. We further confirmed the role of *GmLMM1* in nematode susceptibility by CRISPR-Cas9 mutagenesis. *GmLMM1* encodes a homolog of FER, which regulates a series of cellular processes in *Arabidopsis* (Wang et al., 2020). Consistent with our findings that *GmLMM1* negatively regulates defense response against nematodes, *GmLMM1* has recently been shown to negatively regulate resistance against both bacterial and oomycete pathogens (Wang et al., 2020). Meanwhile, RALF-FER signaling mediated host–RKNs interaction was also studied in the model plants *Arabidopsis* and rice (Zhang et al., 2020a).

Previously, GmLMM1 was reported to participate in PTI signaling network and modulate the ROS production after treatment with the bacterial flg22 or oomycete XEG1 and negatively regulate the resistance to both bacterial and oomycete pathogens (Wang et al., 2020). However, the resistant phenotype was only shown against leaf pathogens, and the role of GmLMM1 in soil-borne disease was unclear in that study. Further, GmLMM1 was found to stabilize the immune complex of FLS2 and BAK1 (Wang et al., 2020), which is similar to FER in Arabidopsis, but the mechanism of which in soybean needs further exploration. Meanwhile, as a homolog of FER, the ligand of GmLMM1 was still unknown. Thus, the role of GmLMM1 in the parasitism of soil-borne pathogens (e.g., nematodes) deserves to be studied, which would facilitate the understanding of the nematode infection. Although *GmLMM1* could play important role in both leaf and root defense, the impact of *GmLMM1* on gene expression remains unknown. In this study, we determined the impact of *GmLMM1* on the overall transcriptional changes. Since JA signaling is closely linked with the parasitism of RKNs (Nahar et al., 2011; Martinez-Medina et al., 2017; Wang et al., 2019), the changes in JA-related genes were considered to be the marker of a successful parasitism. Previous work has shown that FER modulates JA signaling by influencing the protein stability of the transcription factor MYC2, a key regulator of JA signaling (Guo et al., 2018; Zhang et al., 2020a). As the homolog of *FER* in soybean, we found that *GmLMM1* also regulates the JA signaling at the transcriptional level. In addition, *GmLMM1* regulates genes involved in defense responses, cell wall organization, and response to biotic stimulus, which would act on the parasitism of RKNs. Intriguingly, we found that the genes related to hydrogen peroxide catabolic process and the response to oxidative stress were also significantly changed. FER was previously reported to be involved in ROS burst signaling to modulate the reproduction and the bacterial defense (Keinath et al., 2010; Kessler et al., 2010; Yu et al., 2012; Shen et al., 2017; Stegmann et al., 2017), and the infection of RKNs also accompanied with ROS burst (Wang et al., 2019). FER is a scaffold protein facilitating the immune complex formation between FLS2 and BAK1. It has been shown that nematode-induced PTI responses are mediated by BAK1 (Mendy et al., 2017), a coreceptor for LRR-RLKs. Given that nematode does not encode flg22 peptide, the receptor for nematode-associated molecular pattern is unlikely FLS2. It is likely that the soybean FER-like GmLMM1 is also involved in nematode-mediated immune responses with the triggering of ROS burst, but the mechanism needs to be further studied.

The RALF-FER signaling is ubiquitous across the plant kingdom and plays vital roles in plant development, reproduction, flowering, and abiotic and biotic stress (Franck et al., 2018; Zhang et al., 2020b). Recently, RALF-FER signaling was shown to be hijacked by nematode secreted RALF-like peptides, to modulate the interaction between host and RKNs (Zhang et al., 2020a). Precisely, *Mi*RALF1/3 secreted from RKN *M. incognita* bind to FER, triggered MAPK phosphorylation, and destabilized MYC2 protein (Zhang et al., 2020a). In this study, GmLMM1 and GmLLG2 were found to finely match the 3D structure of RALF23-LLG2-FER, suggesting that the RALF perception mechanism may be conserved across different plant species. However, our study showed that only *Mi*RALF1 and *At*RALF23 could bind to GmLMM1, but not *At*RALF1 and *Mi*RALF3. This is rather extraordinary and it is possible that the evolution of plant and nematode RALFs determined various RALF ligand strategies to activate different FER homologs in multiple plant species. The interaction of GmRALFs from soybean with GmLMM1 and the difference of binding affinity between GmRALFs and *Mi*RALFs to GmLMM1 need further investigations.

We detected the relationship of natural variation of *GmLMM1* with the resistance against *M. incognita*. Interestingly, the varieties belong to H3 or H5 showed enhanced resistance against *M. incognita*, and the SNP of 1075A/C leading to the amino acid mutation of N359H seems to be vital for the natural resistance of GmLMM1 (Figure 5; Supplementary Figure S1B). We have predicted the model of GmLMM1^ECD^ and GmLMM1^ECDN359H^ by AlphaFold, which showed no significant differences based on the current structure. We failed to obtain the direct evidence that the mutation of N359H affect the binding of RALF23 to GmLMM1 (Supplementary Figure S8). Previously, RALF1 was found to bind the second malectin-like domain of the FER extracellular domain, in which the N359 is located (Feng et al., 2018). Moreover, the N359 is nearly located in the linker between α helix and β folding domain, which is the key binding region of FER to LLG1 (Li et al., 2015). Therefore, the AlphaFold structure prediction probably cannot fully explain the binding of MiRALF1 to GmLMM1. Thus, to reveal the role of N359H in the structure of RALF-GmLMM1-LLG2 complex, the new crystal structure needs to be further analyzed. Notably, the single point mutation of L407H leads to resistance of *Gmlmm1-2* to RKNs, that L407 located in the β folding domain, which supposed that the interaction of FER with LLG1 might be essential to the resistance against RKNs, and the detailed mechanism needs to be further studied. Meanwhile, the SNPs of 25A/C and 119C/A lead to the amino acid mutation of T9P and T40N, which were not located in the RALF-binding domain, contributing to no significant difference in resistance to RKN (Liu et al., 2018; Xiao et al., 2019). In the last decades, natural variation in the resistance genes was found to be the conventional approaches to defense the pathogens in plants (Broekgaarden et al., 2011). Precisely, the natural variation leading to the absence or amino acid change of RPS4/RRS1 improved the resistance of host to *Pseudomonas syringae* pv. tomato, *Ralstonia solanacearum*. and *Colletotrichum higginsianum* (Narusaka et al., 2017). Moreover, the nature of resistance to cyst nematode was revealed that the SNP of *SHMT* conferred resistance or susceptibility (Liu et al., 2012). Therefore, understanding and exploring the natural resistance of soybean to RKN is increasingly important for the long-term management, making it an excellent entry point for resistance breeding.

In summary, crosskingdom signaling consisting of nematode-encoded RALF-likes and plant receptors GmLMM1 and FER play important roles in the plant resistance against RKNs, making it an excellent target for resistant breeding and an excellent entry point for studying the interactions between RKNs and theirs hosts.

## Data Availability Statement

RNA-seq data generated in this study have been deposited to NCBI under accession number PRJNA763990 (https://www.ncbi.nlm.nih.gov/bioproject/PRJNA763990).

## Author Contributions

FY, XF, and DWu designed the research. DWa, XZ, and JC performed the experiments. XZ and DWa wrote the manuscript with input from other co-authors. All authors contributed to the article and approved the submitted version.

## Funding

This work was supported by a starting grant from Hunan University (531118010507) to DWu, Key Research Program of the Chinese Academy of Sciences (Grant Nos. ZDRW-ZS-2019-2-02) to XF and China Postdoctoral Science Foundation funded projects (No. 2021M701091) to XZ.

## Conflict of Interest

The authors declare that the research was conducted in the absence of any commercial or financial relationships that could be construed as a potential conflict of interest.

## Publisher's Note

All claims expressed in this article are solely those of the authors and do not necessarily represent those of their affiliated organizations, or those of the publisher, the editors and the reviewers. Any product that may be evaluated in this article, or claim that may be made by its manufacturer, is not guaranteed or endorsed by the publisher.

## Supplementary Material

Supplementary Figure S1 Alignment of GmLMM1 and FER. **(A)** *Gm*LMM1 protein domain. Two corresponding mutated sites in *rmi1* and *rmi2* are indicated. **(B)** Alignment of extracellular domain (ECD) sequences of GmLMM1 and FER.

Supplementary Figure S2 Neighbor-joining phylogenetic analysis of GmLMM1 and Arabidopsis *Cr*RLK1L family.

Supplementary Figure S3 Motif-GO enrichment analysis. **(A)** Motif-GO enrichment analysis based on the promoters of the DEGs under the infection with *M. incognita*. **(B)** GO enrichment analysis based on the *p*-values for similarly expressed genes corresponding to the DEGs of **(A)**. The numbers in brackets represent the number of genes. GO category red stained was involved in RKN resistance. **(C)** Motif-GO enrichment analysis based on the promoters of the DEGs with the mutation of GmLMM1. **(D)** GO enrichment analysis based on the *p*-values for similarly expressed genes corresponding to the DEGs of **(C)**. The numbers in brackets represent the number of genes. GO category red stained was involved in RKN resistance.

Supplementary Figure S4 Neighbor-joining phylogenetic analysis of GmRALFs and Arabidopsis RALF1/23.

Supplementary Figure S5 GST pull-down assay of GmRALFs with GmLMM1. GST-GmLMM1^ECD^ pulled down the GmRALFs. Anti-His antibody and anti-GST antibody were used to detect the GmRALFs and GmLMM1, respectively.

Supplementary Figure S6 Nematode numbers of Col-0 and *fer-4* plant roots treated with exogenous 1 μM MiRALF1 and AtRALF1 peptides at 3 dpi. Data are presented as the mean ±S.D., *n* ≥ 10; Student's *t*-test (^**^ *p* < 0.01; n.s., not significant) and RALF-like peptides were heterologously expressed in *E. coli*.

Supplementary Figure S7 Nematode numbers per gram root of the cultivars with different *GmLMM1* haplotypes grouped based on the three nonsynonymous SNPs. **(A)** SNP1 of 25A/C (p. T9P) showed no significance in nematode numbers. **(B)** SNP2 of 119C/A (p. T40N) showed no significance in nematode numbers. **(C)** SNP3 of 1075A/C (p. N359H) showed a significance in nematode numbers. Data are presented as the mean ± S.D., *n* ≥ 10; Student's *t*-tests.

Supplementary Figure S8 3D structure analysis of GmLMM1. **(A)** The 3D structure of the AtRALF23-GmLLG2/GmLMM1^ECD^ complex. AtRALF23, GmLLG2, and GmLMM1^ECD^ are colored light green, blue, and clay bank, respectively. The black frame indicated the position of N359. **(B)** The 3D structure of GmLMM1^ECD^ and GmLMM1^ECDN359H^ predicted by AlphaFold. The black frame indicated the position of N359. **(C)** The difference of N359 and H359 in the GmLMM1^ECD^ 3D structure.

Supplementary Table S1 Primers used in this study.

Supplementary Table S2 Comparison of the three nonsynonymous SNPs in the coding region of *GmLMM1* from the 232 varieties of cultivated soybeans.
