# Supplementary figures and images for: Nematode RALF-Like 1 Targets Soybean Malectin-Like Receptor Kinase to Facilitate Parasitism
Source: Front Plant Sci. 2021 Dec 17;12:775508. doi: 10.3389/fpls.2021.775508 (PMC8719587; doi:10.3389/fpls.2021.775508)

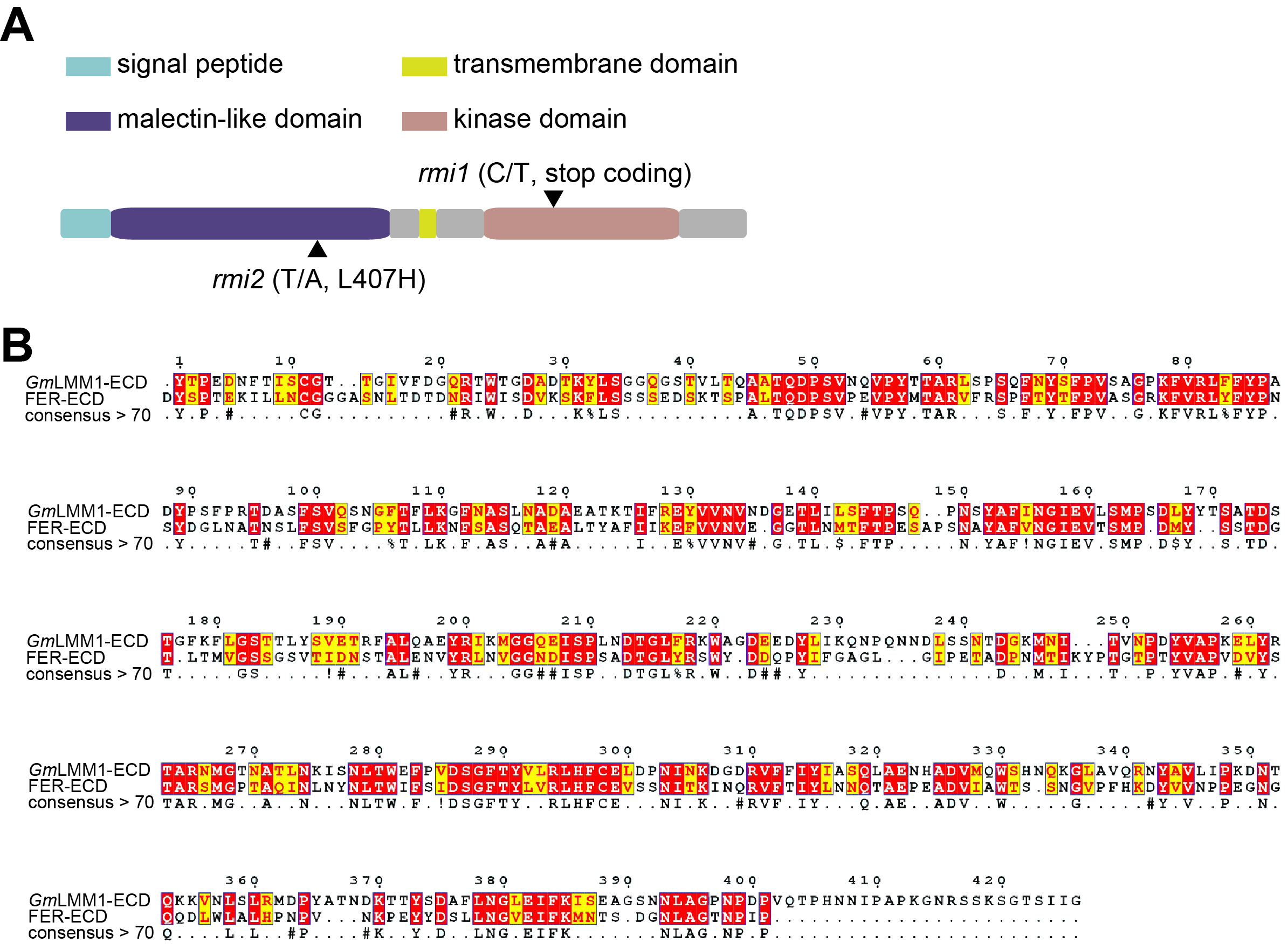

Supplement: Supplementary Figure S1 — Alignment of GmLMM1 and FER. (A) GmLMM1 protein domain. Two corresponding mutated sites in rmi1 and rmi2 are indicated. (B) Alignment of extracellular domain (ECD) sequences of GmLMM1 and FER. [file Image_1.JPEG]

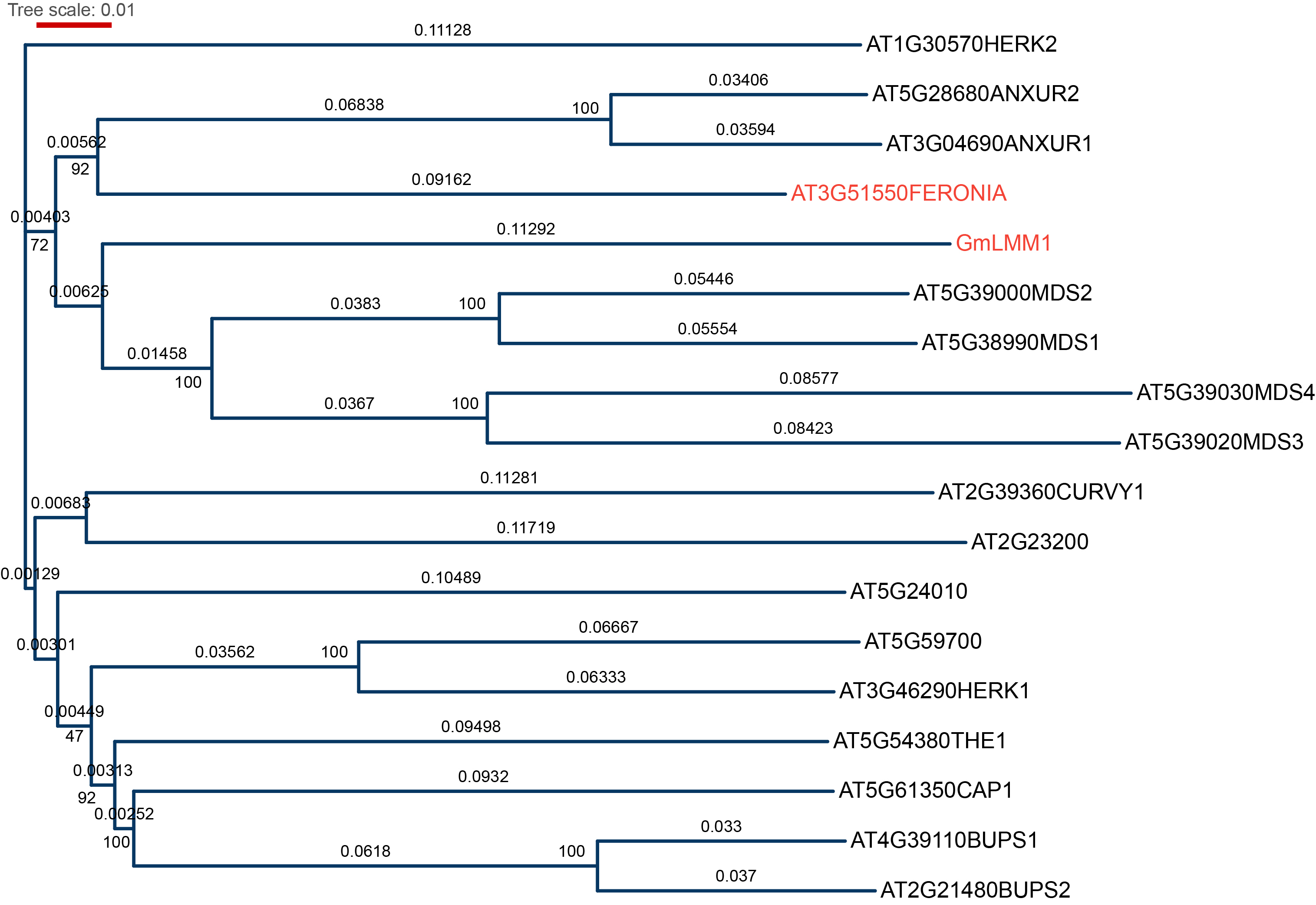

Supplement: Supplementary Figure S2 — Neighbor-joining phylogenetic analysis of GmLMM1 and Arabidopsis CrRLK1L family. [file Image_2.JPEG]

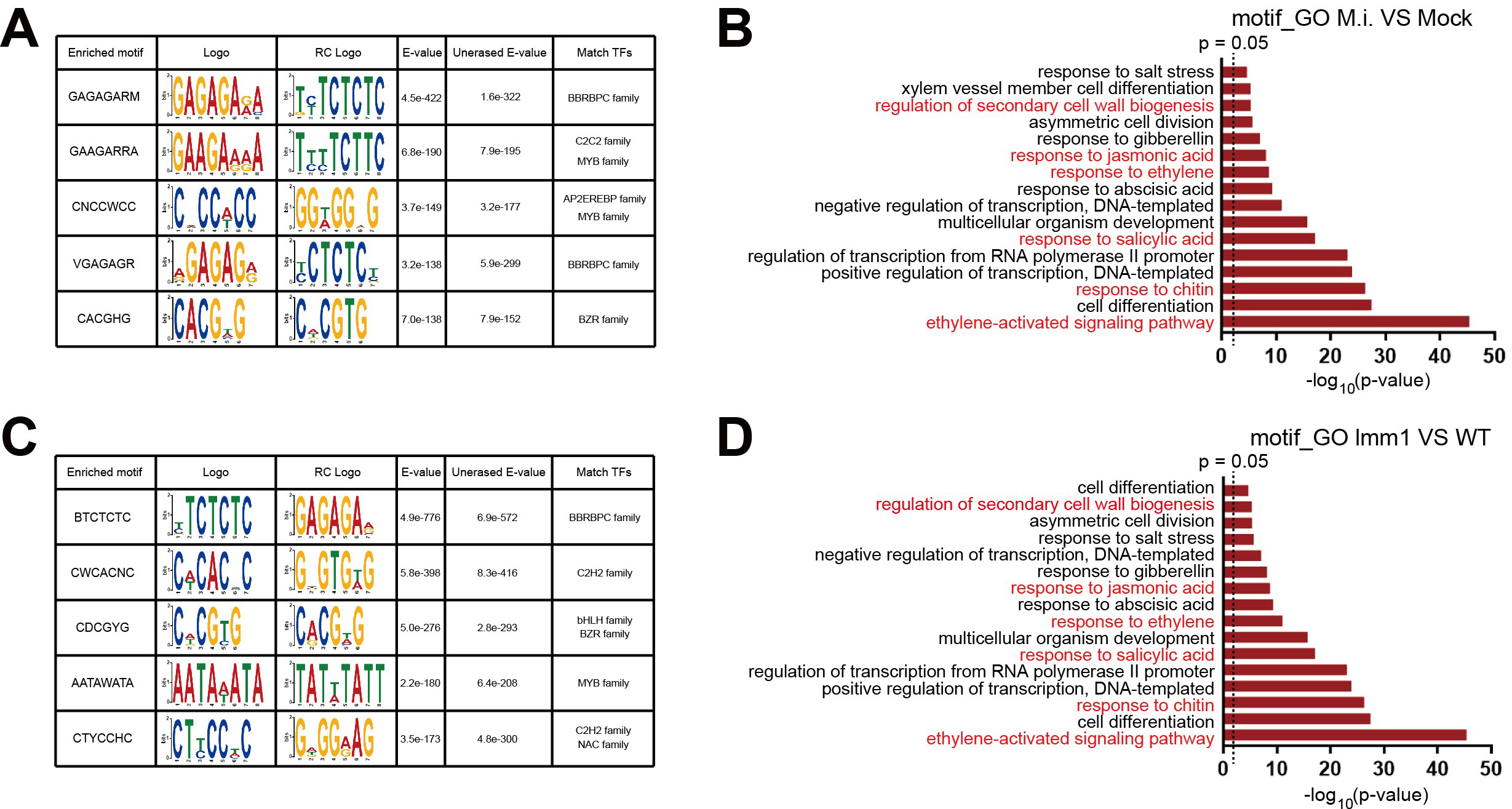

Supplement: Supplementary Figure S3 — Motif-GO enrichment analysis. (A) Motif-GO enrichment analysis based on the promoters of the DEGs under the infection with M. incognita. (B) GO enrichment analysis based on the p-values for similarly expressed genes corresponding to the DEGs of (A). The numbers in brackets represent the number of genes. GO category red stained was involved in RKN resistance. (C) Motif-GO enrichment analysis based on the promoters of the DEGs with the mutation of GmLMM1. (D) GO enrichment analysis based on the p-values for similarly expressed genes corresponding to the DEGs of (C). The numbers in brackets represent the number of genes. GO category red stained was involved in RKN resistance. [file Image_3.JPEG]

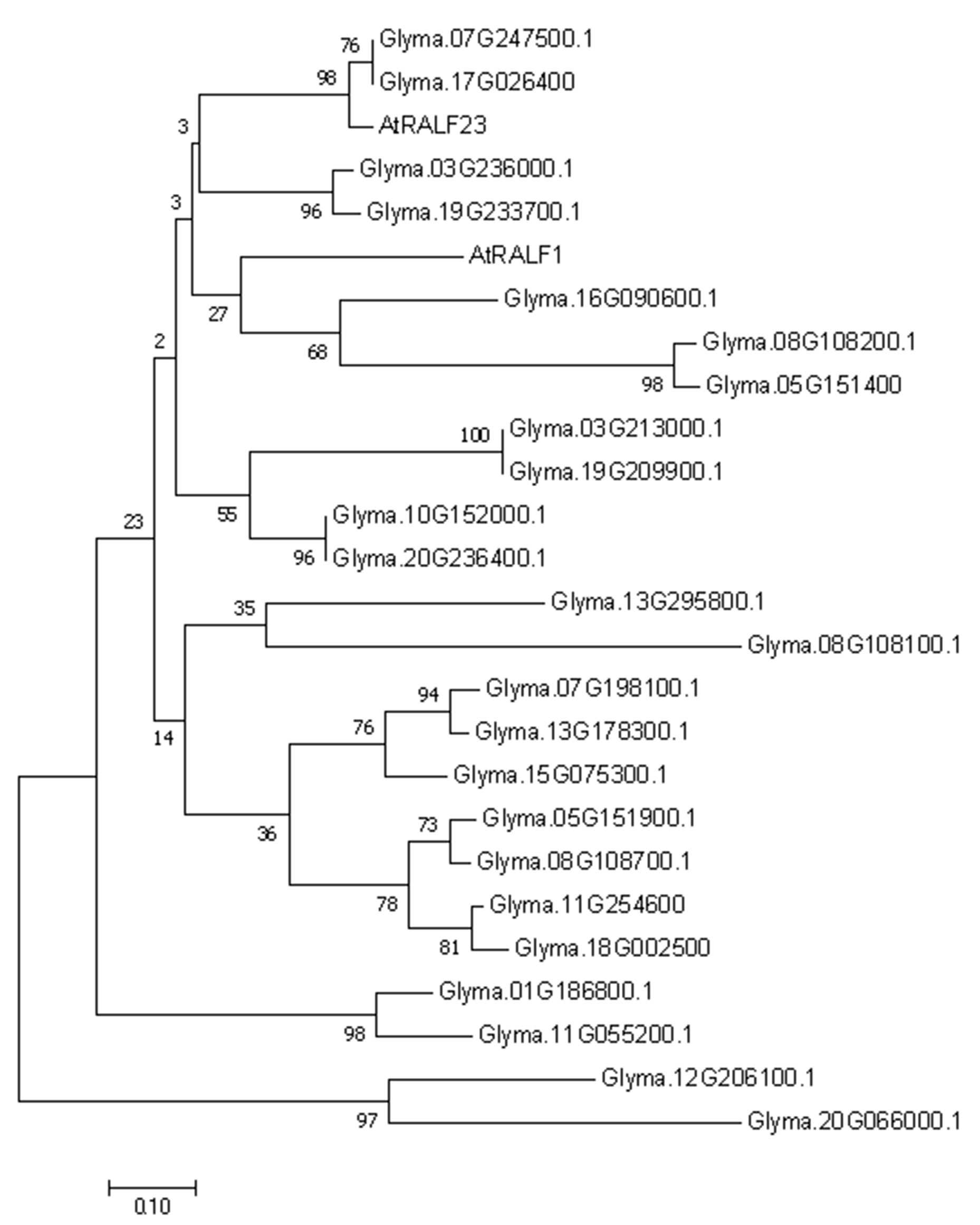

Supplement: Supplementary Figure S4 — Neighbor-joining phylogenetic analysis of GmRALFs and Arabidopsis RALF1/23. [file Image_4.TIFF]

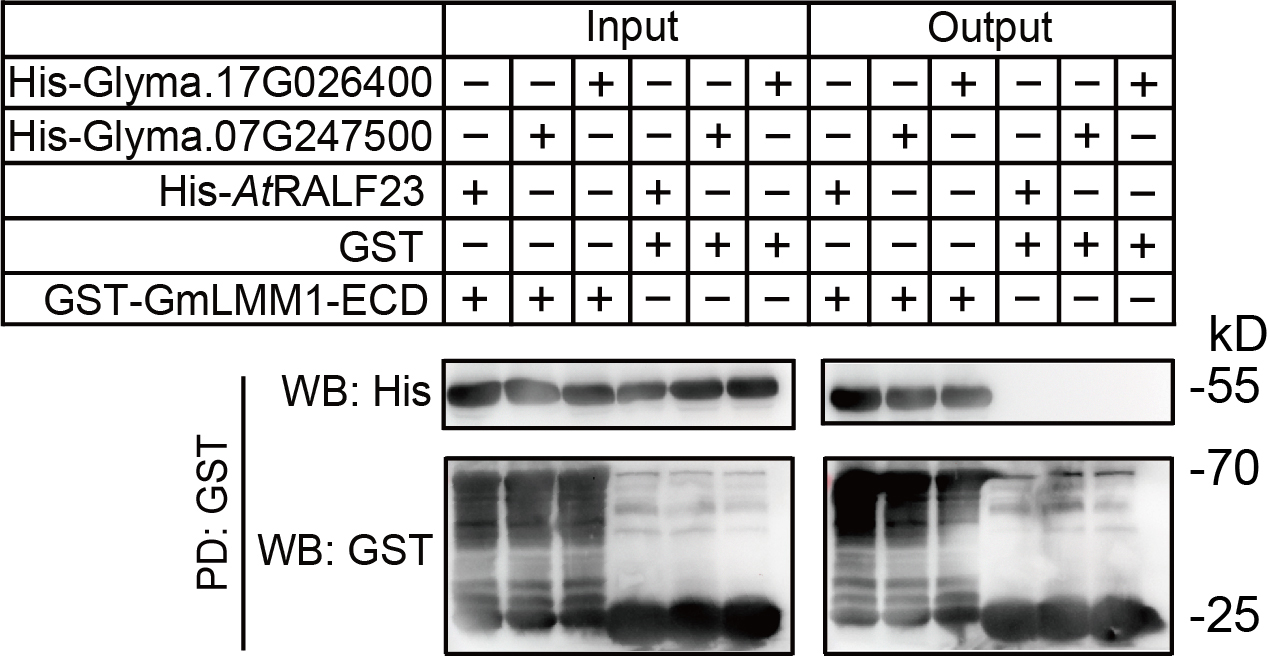

Supplement: Supplementary Figure S5 — GST pull-down assay of GmRALFs with GmLMM1. GST-GmLMM1ECD pulled down the GmRALFs. Anti-His antibody and anti-GST antibody were used to detect the GmRALFs and GmLMM1, respectively. [file Image_5.JPEG]

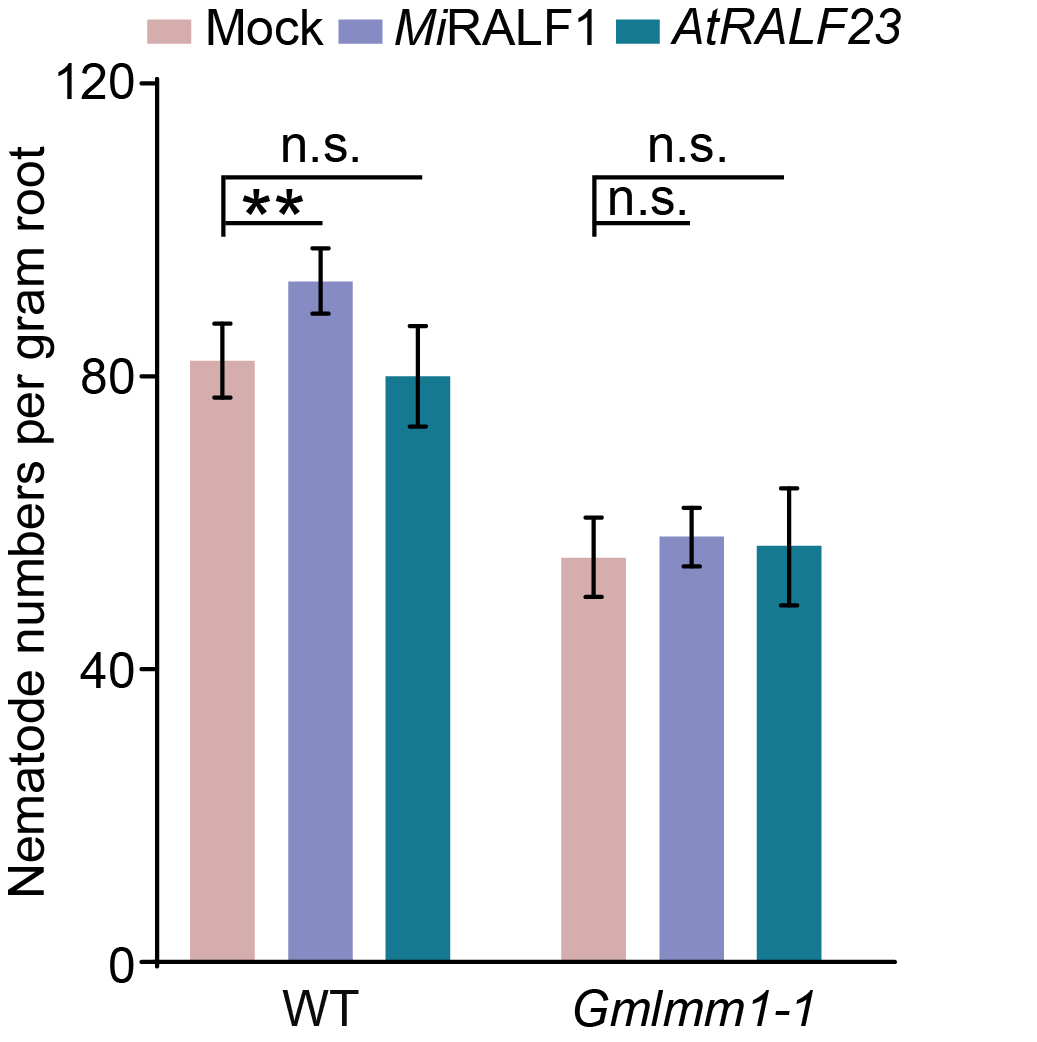

Supplement: Supplementary Figure S6 — Nematode numbers of Col-0 and fer-4 plant roots treated with exogenous 1 μM MiRALF1 and AtRALF1 peptides at 3 dpi. Data are presented as the mean ±S.D., n ≥ 10; Student's t-test (** p < 0.01; n.s., not significant) and RALF-like peptides were heterologously expressed in E. coli. [file Image_6.JPEG]

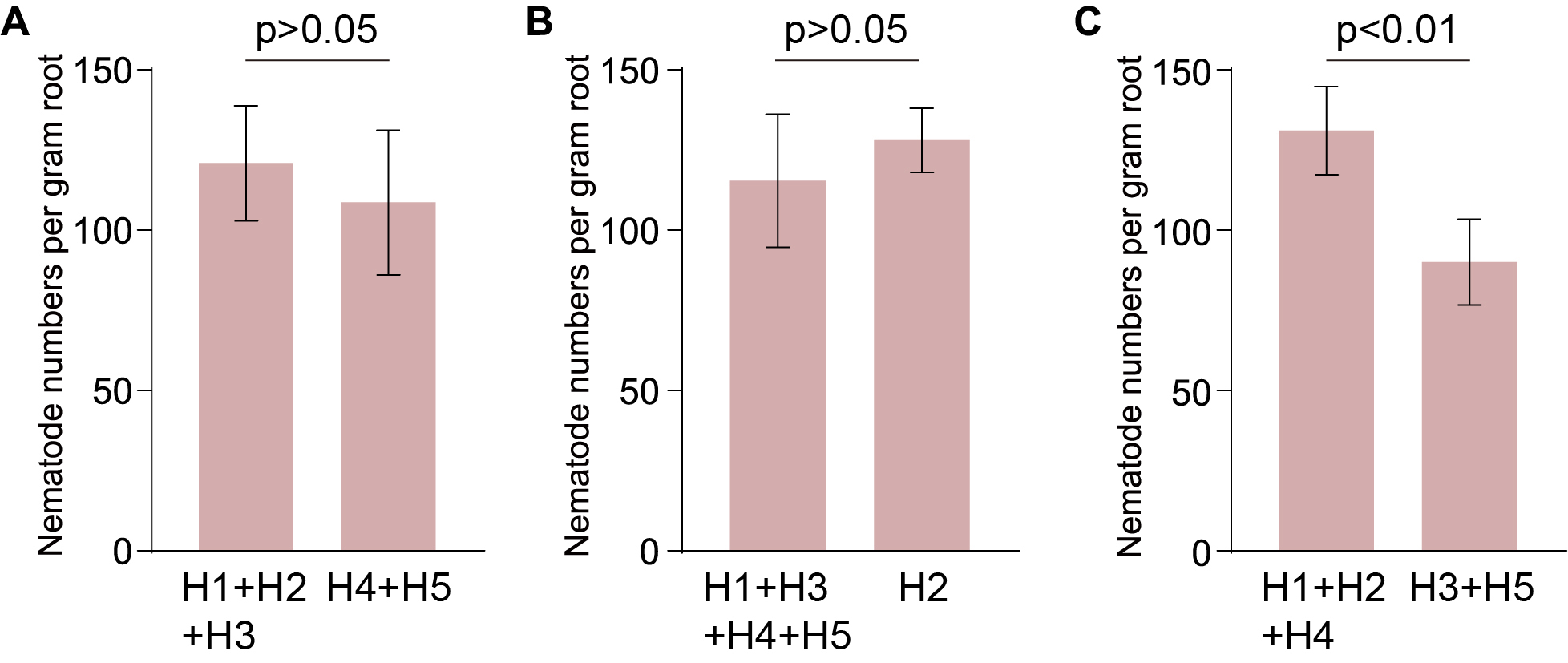

Supplement: Supplementary Figure S7 — Nematode numbers per gram root of the cultivars with different GmLMM1 haplotypes grouped based on the three nonsynonymous SNPs. (A) SNP1 of 25A/C (p. T9P) showed no significance in nematode numbers. (B) SNP2 of 119C/A (p. T40N) showed no significance in nematode numbers. (C) SNP3 of 1075A/C (p. N359H) showed a significance in nematode numbers. Data are presented as the mean ± S.D., n ≥ 10; Student's t-tests. [file Image_7.JPEG]

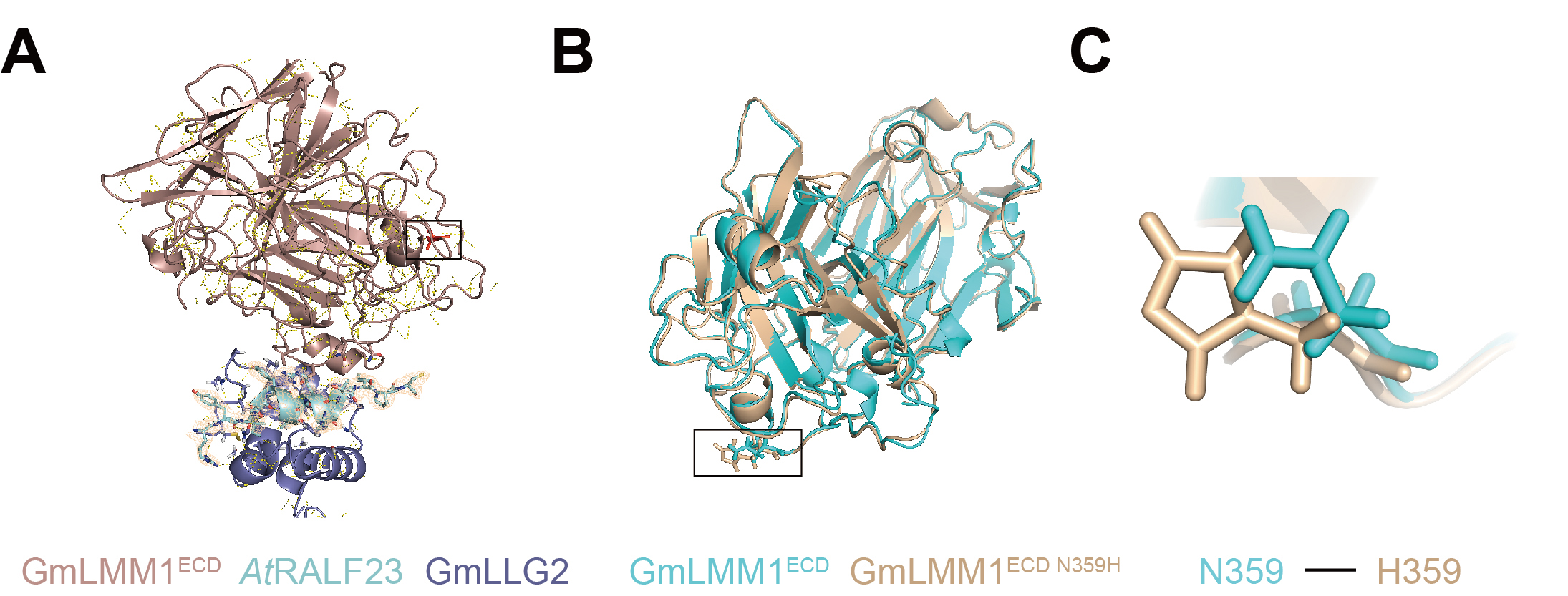

Supplement: Supplementary Figure S8 — 3D structure analysis of GmLMM1. (A) The 3D structure of the AtRALF23-GmLLG2/GmLMM1ECD complex. AtRALF23, GmLLG2, and GmLMM1ECD are colored light green, blue, and clay bank, respectively. The black frame indicated the position of N359. (B) The 3D structure of GmLMM1ECD and GmLMM1ECDN359H predicted by AlphaFold. The black frame indicated the position of N359. (C) The difference of N359 and H359 in the GmLMM1ECD 3D structure. [file Image_8.JPEG]
